# An emerging recombinant human enterovirus 71 responsible for the 2008 outbreak of Hand Foot and Mouth Disease in Fuyang city of China

**DOI:** 10.1186/1743-422X-7-94

**Published:** 2010-05-12

**Authors:** Yan Zhang, Zhen Zhu, Weizhong Yang, Jun Ren, Xiaojuan Tan, Yu Wang, Naiying Mao, Songtao Xu, Shuangli Zhu, Aili Cui, Yong Zhang, Dongmei Yan, Qun Li, Xiaoping Dong, Jing Zhang, Yueping Zhao, Junfeng Wan, Zijian Feng, Junling Sun, Shiwen Wang, Dexin Li, Wenbo Xu

**Affiliations:** 1 Key Laboratory for Molecular Virology & Genetic Engineering, National Institute for Viral Disease Control and Prevention, Chinese Center for Disease Control and Prevention, Beijing 100052, China; 2 Chinese Center for Disease Control and Prevention, Beijing 100050, China; 3 Anhui Provincial Center for Disease Control and Prevention, Hefei 230001, China; 4 Office for Disease Control and Emergency Response, Chinese Center for Disease Control and Prevention, Beijing 100050, China; 5 Fuyang prefecture Center for Disease Control and Prevention, Fuyang 236000, China

## Abstract

Hand, foot and mouth disease (HFMD), a common contagious disease that usually affects children, is normally mild but can have life-threatening manifestations. It can be caused by enteroviruses, particularly Coxsackieviruses and human enterovirus 71 (HEV71) with highly variable clinical manifestations. In the spring of 2008, a large, unprecedented HFMD outbreak in Fuyang city of Anhui province in the central part of southeastern China resulted in a high aggregation of fatal cases. In this study, epidemiologic and clinical investigations, laboratory testing, and genetic analyses were performed to identify the causal pathogen of the outbreak. Of the 6,049 cases reported between 1 March and 9 May of 2008, 3023 (50%) were hospitalized, 353 (5.8%) were severe and 22 (0.36%) were fatal. HEV71 was confirmed as the etiological pathogen of the outbreak. Phylogenetic analyses of entire VP1 capsid protein sequence of 45 Fuyang HEV71 isolates showed that they belong to C4a cluster of the C4 subgenotype. In addition, genetic recombinations were found in the 3D region (RNA-dependent RNA polymerase, a major component of the viral replication complex of the genome) between the Fuyang HEV71 strain and Coxsackievirus A16 (CV-A16), resulting in a recombination virus. In conclusion, an emerging recombinant HEV71 was responsible for the HFMD outbreak in Fuyang City of China, 2008.

## Background

The first HEV71 infection was reported in 1969 in the US [1]. Although HFMD is usually a mild disease, several HEV71 outbreaks associated with severe neurological complications have been reported in Western Pacific countries or regions, including Malaysia in 1997; Taiwan in 1998, 2000 and 2001; Australia in 1999; Singapore in 2000; Japan in 1997 and 2000; and Shandong of China in 2007 [2-8].

Human enterovirus 71 (HEV71) is one of the member in HEV-A species of *Enterovius *genus in the family *Picornaviridae*. The genome of HEV71 consists of a single-stranded positive-sense RNA of approximately 7400 nucleotides. The viral genome contains a 5'- and 3'-untranslated regions (UTRs) which are essential for viral RNA replication. The genome is translated as a single large polyprotein that is composed of four capsid proteins, VP1 to VP4, and seven nonstructural proteins, 2A, 2B, 2C, 3A, 3B, 3C, and 3D. VP1 to VP4 capsid proteins were encoded by P1 region. The P2 and P3 regions encode nonstructural proteins and the RNA-dependent RNA polymerase, 3D, is a major component of the viral replication complex [9].

Sporadic epidemic or outbreaks of HEV71 infection had occurred in mainland of China since 1995[8,10,11], but the highly aggregated cases of rapidly fatal pediatric infections occurred in the Fuyang HFMD outbreak in 2008 is unprecedented. The identification of the causal pathogen is crucial for preventing disease spread, reducing fatality rate, and better understanding the pathogenic mechanism underlying disease severity. This study reported the investigation and the etiological identification of the Fuyang HFMD outbreak in 2008. And the genetic recombination event was also found between the Fuyang HEV71 isolates and CV-A16.

## Results

### Cases and epidemiology

**Figure 1:**
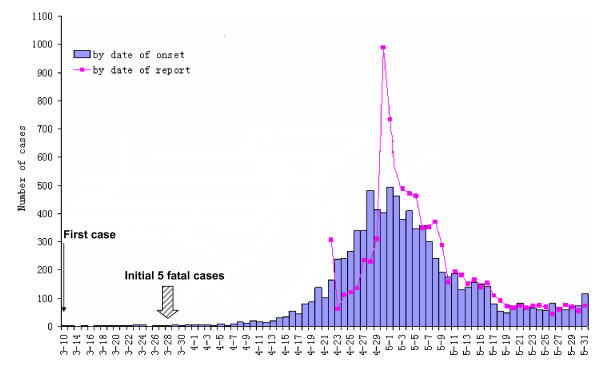
**Distribution of hand, foot, and mouth disease (HFMD) patients in Fuyang City, Anhui province, by date**. By 31 May 2008, the outbreak had affected 6883 children (This study focused on the outbreak between March 1 and May 9 of 2008, which included 6049 reported cases). The bold slipped arrows indicated the death dates of initial 5 fatal cases.

This study focused on the outbreak between 1 March and 9 May, 2008. A total of 6,049 HFMD cases were reported from local healthcare facility in Fuyang city (Figure 1), of which 3,023 (50%) were hospitalized, 353 (6%) were severe and 22 (0.4%) were fatal. The incidence rate was 70/100,000; while the fatality rate was 0.4%. The gender ratio of the epidemics was 1.9:1, with 3,938 male and 2,111 female cases. The age ranged from 28 days to 18 years, with 78% of the cases < 3 years old. All of the 22 fatal cases were < 3 years old, and the youngest was 3 months.

### Case definitions

The mild HFMD outpatients were defined as patients with fever and vesicular lesions on their palms, feet and mouth. Severe cases usually presented with neurologic complications, which were defined as having two of following clinical manifestations: brainstem encephalitis or aseptic meningitis; continuous high fever (temperature of at least 38°C); weakness, vomiting, irritability, myoclonus and acute flaccid paralysis; pulmonary edema or hemorrhage, heart and lung failure. Encephalitis, aseptic meningitis, pulmonary edema, pulmonary hemorrhage, acute flaccid paralysis, myocarditis were characterized by the same definition as described previously [2].

### Clinical description

General clinical symptoms of the Fuyang HFMD outbreak included rash, fever, general malaise, cough, vomiting and neurologic complications (such as encephalitis, aseptic meningitis, or acute flaccid paralysis). The analysis of the clinical characteristics of 15 out of 22 fatal cases showed that these cases had an acute onset of fever, sore throat, and myalgia (influenza-like illness) without catarrhal syndrome, but the rash was rare. The conditions of most of cases subsequently deteriorated, in which the patients developed tachypnea, cyanosis, and some presented seizures with white or pink foaming at the mouth (Table 1). Most hospitalized cases were initially diagnosed as severe pneumonia, some patients died within 1-5 days after onset.

**Table 1:** Clinical symptoms of fatal HFMD cases in Fuyang hospital (n = 15).

Clinical symptom	Number of cases	Proportion (%)
Fever	15	100
Tachypnea	14	93.3
Oral cyanosis	12	80
Pink foaming at the mouth	9	60
Coughing	7	46.7
Vomiting	8	53.3
Myoclonic twitching	5	33.3
Tiny pink rash on palm, sole	2	13.3
Nasal discharge	2	13.3
Stiff neck	2	13.3

### Laboratory identification of the etiologic pathogen of the outbreak

Since most of fatal or severe cases were characterized as respiratory disease syndrome, direct RT-PCR were performed on a total of 89 specimens (including blood sera, throat swabs and autopsy tissue samples) collected from fatal and severe cases to detect for seasonal influenza, avian influenza A/H5N1, Severe Acute Respiratory Syndrome (SARS), RSV, Adeno virus, and other respiratory bacteria microorganism, and all the results were negative. As enterovirus infection could also induce the respiratory symptoms, the specimens were also tested using primers for pan-enterovirus, HEV71, CV-A16 and Echovirus. Positive results were obtained only for pan-enterovirus and HEV71. Further investigation using sequence determination and BLAST sequence analysis revealed that all of the sequences had high homology (95.1%) with HEV71 (strain E2004104-TW-CDC, accession number EF373576). To further confirm the possible etiological pathogen, additional 121 more clinical specimens were collected for the detection of HEV71 and CV-A16 using direct RT-PCR.

Thus, a total of 210 specimens collected from 13 fatal cases, 99 severe cases and 39 mild cases, respectively, had been tested for HEV71 and CV-A16 using direct RT-PCR. Six (46%) of 13 fatal cases, 36 (36%) of 99 severe cases, 17 (44%) of 39 mild cases were positive for HEV71 (Table 2). None were positive for CV-A16 infection. Gene sequencing studies of HEV71 showed that sequences from fatal, severe and mild cases exhibited high homology.

**Table 2:** Case classification and HEV71 positive number (rate) by RT-PCR or virus isolation.

		HEV71		Other Enterovirus	
			
Case Classification	No. of Cases	direct RT-PCR	virus Isolation	direct RT-PCR for CV-A16	virus Isolation
Fatal cases	13	6(46%)	6(46%)	0	0
Severe cases	99	36(36%)	26(26%)	0	1(E9),1(CV-A4)
Mild outpatients	39	17(44%)	13(33%)	0	1(E9),1(CV-B3), 1(CV-A9)
Total	151	59(39%)	45(30%)	0	5

Note: E9: Echovirus 9; CV-A4: Coxackievirus A4; CV-B3: Coxackievirus B3; CV-A9:Coxackievirus A9. All the positive PCR fragments and isolates were confirmed by sequencing.

All of the 210 specimens were separately cultured in RD and HEp-2 cell lines. A total of 50 enterovirus isolates were obtained from clinical specimens: 34 of 132 (26%) throat swabs, 7 of 12 (58%) stool samples, 3 of 4 (75%) vesicular fluid, 3 of 22 (14%) anus swab, 1 of 2 (50%) antemortem oral efflux, and 2 of 23 (9%) autopsy lung tissue. No virus was isolated from CSF and other autopsy tissue samples. By RT-PCR and sequence determination, 45/50 (90%) of the enteroviruses were identified as HEV71. In addition, 2 Echovirus 9 (E9), 1 Coxsackievirus A4 (CV-A4), 1 Coxackievirus A9 (CV-A9) and 1 Coxackievirus B3 (CV-B3) were isolated and identified. No CV-A16 virus was found.

Taken together, the results showed that HEV71 was associated with the Fuyang HFMD outbreak in 2008.

### Phylogenetic analysis of entire VP1 region of HEV71

The entire VP1 sequences of 45 HEV71 isolates from 6 fatal cases, 26 severe cases and 13 mild outpatients were used for phylogenetic analysis(data now shown). The nucleotide and the amino acid homologies among the Fuyang isolates were 96.4%-100% and 98.7%-100%, respectively. The average nucleotide homologies within fatal, severe and mild cases were 99.3%, 99.7% and 98.9%, respectively. Based on the VP1 gene, the nucleotide and amino acid homology between fatal or severe cases and mild cases were 96.4%-100% and 98.7%-100%, respectively. No significant nucleotide and amino acid differences in the VP1 region were found between severe or fatal cases and mild cases.

**Figure 2:**
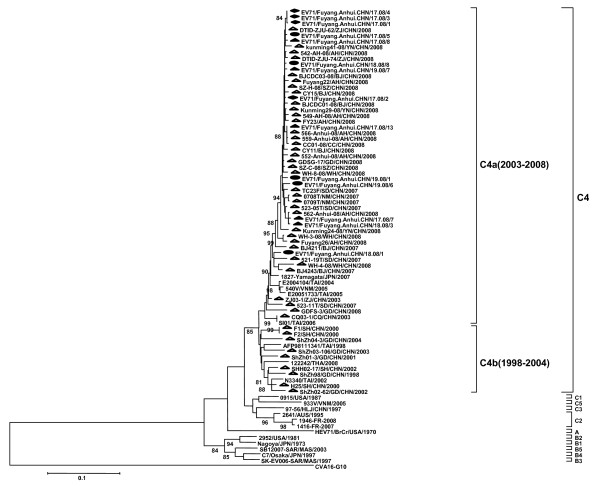
**Phylogenetic dendrogram were drawn on the basis of the 891-nt sequence of entire VP1 gene using the Neighbor-Joining method of MEGA software (version 4.0) for 14 selected Fuyang representative isolates and other HEV71 strains of known subgenotypes listed in Table 1**. The bootstrap values from 1000 pseudoreplicates for major lineages within the tree are shown as percentages. The prototype CVA16 strain (G-10) was used as outgroup. Solid diamond indicated the fatal cases; Solid triangle indicated the severe cases; Solid round indicated the outpatients; Triangle indicated the HEV71 sequences from other provinces in China between 1997-2008 available in GenBank database.

To determine the molecular epidemiology of the Fuyang HEV71 isolates, a phylogenetic dendrogram was constructed with 14 representative Fuyang HEV71 isolates (which were selected based on the case classification and their nucleotide divergence: 4 fatal cases, 8 severe cases, 2 mild cases), 43 HEV71 strains available in the GenBank isolated from 12 provinces in mainland of China between 1997-2008, and 18 international HEV71 strains that represented all 11 known genotypes or subgenotypes (A, B1-B5, C1-C5), and 2 HEV71 strains from 2 fatal French cases of 2007-2008 (Table 3, Figure. 2). It showed that the Fuyang HEV71 isolates belonged to cluster C4a of the subgenotype C4, which was similar to the HEV71 sequences isolated from the mainland of China during 2003-2008 (including the sequences from Shandong Province in 2007 Linyi HFMD outbreak(Zhang, 2009 #233)).

**Table 3:** Entire VP1 gene nucleotide sequences of the HEV71 strains used to generate the HEV71 phylogenetic dendrograms.

Strain	Source	**GenBank No**.	Strain	Source	**GenBank No**.
HEV71/BrCr/USA/70	GenBank	U22521	TC23F/SD/CHN/2007	GenBank	EU753417
CVA16/G-10	GenBank	U05876	CY11/BJ/CHN/2008	GenBank	FJ469153
Nagoya/JPN/73	GenBank	AB059813	CY15/BJ/CHN/2008	GenBank	FJ469154
2952/USA/81	GenBank	AF135888	DTID-ZJU-62/ZJ/CHN/2008	GenBank	FJ158600
0915/USA/87	GenBank	AF009549	DTID-ZJU-74/ZJ/CHN/2008	GenBank	FJ158600
2641/AUS/95	GenBank	AF135947	GDFS-3/GD/CHN/2008	GenBank	FJ194964
SK-EV006-SAR/MAS/97	GenBank	AB059819	GDSG-17/GD/CHN/2008	GenBank	FJ194965
C7/Osaka/JPN/97	GenBank	AB059818	FY23/AH/CHN/2008	GenBank	EU812515
SB12007-SAR/MAS/03	GenBank	AY905548	Fuyang22/AH/CHN/2008	GenBank	EU913466
933V/VNM/05	GenBank	AM49061	Fuyang26/AH/CHN/2008	GenBank	EU913468
540V/VNM/05	GenBank	AM490151	WH-4-08/WH/CHN/2008	GenBank	FJ765433
SI01/THA/06	GenBank	EF203407	WH-3-08/WH/CHN/2008	GenBank	FJ765432
1827-Yamagata/JPN/07	GenBank	AB433890	Kunming24-08/YN/CHN/2008	GenBank	FJ765425
122242/THA/08	GenBank	FJ151494	562AH-08/AH/CHN/2008	GenBank	FJ765420
AFP98111341/TAI/98	GenBank	DQ841953	WH-8-08/WH/CHN/2008	GenBank	FJ765434
N3340/TAI/98	GenBank	EU131776	SZ-C-08/SZ/CHN/2008	GenBank	FJ765429
E2004104/TAI/04	GenBank	DQ841964	552-AH-08/AH/CHN/2008	GenBank	FJ765418
E20051733/TAI/05	GenBank	DQ841971	CC01-08/CC/CHN/2008	GenBank	FJ765424
1946-FR-2008	GenBank	FJ824736	559-AH-08/AH/CHN/2008	GenBank	FJ765419
1416-FR-2007	GenBank	FJ824734	549-AH-08/AH/CHN/2008	GenBank	FJ765417
97-56/HLJ/CHN/97	GenBank	AB115494	Kunming29-08/YN/CHN/2008	GenBank	FJ765426
ShZh98/GD/CHN/98	GenBank	AF302996	BJCDC01-08/BJ/CHN/2008	GenBank	FJ765424
H25/SH/CHN/2000	GenBank	AB115492	SZ-H-08/SZ/CHN/2008	GenBank	FJ765430
F1/SH/CHN/2000	GenBank	AB115490	BJCDC03-08/BJ/CHN/2008	GenBank	FJ765423
F2/SH/CHN/2000	GenBank	AB115491	EV71/Fuyang.Anhui.CHN/17.08/1	This study	EU703812
ShZh01-3/CHN/2001	GenBank	AY895132	EV71/Fuyang.Anhui.CHN/17.08/2	This study	EU703813
SHH02-17/SH/CHN/2002	GenBank	AY547500	EV71/Fuyang.Anhui.CHN/17.08/3	This study	EU703814
ShZh02-62/CHN/2002	GenBank	AY895136	EV71/Fuyang.Anhui.CHN/17.08/5	This study	GQ121418
ZJ03-1/ZJ/CHN/2003	GenBank	AY905614	EV71/Fuyang.Anhui.CHN/17.08/7	This study	GQ121420
CQ03-1/CQ/CHN/2003	GenBank	AY547501	EV71/Fuyang.Anhui.CHN/17.08/8	This study	GQ121421
ShZh03-106/GD/CHN/2003	GenBank	AY895138	EV71/Fuyang.Anhui.CHN/17.08/4	This study	GQ121423
ShZh04-3/GD/CHN/2004	GenBank	AY895142	EV71/Fuyang.Anhui.CHN/19.08/7	This study	GQ121424
0708T/NM/CHN/2007	GenBank	EU910861	EV71/Fuyang.Anhui.CHN/18.08/1	This study	GQ121427
0709T/NM/CHN/2007	GenBank	EU910862	EV71/Fuyang.Anhui.CHN/17.08/13	This study	GQ121428
BJ4211/BJ/CHN/2007	GenBank	EU024958	EV71/Fuyang.Anhui.CHN/18.08/3	This study	GQ121431
BJ4243/BJ/CHN/2007	GenBank	EU019910	EV71/Fuyang.Anhui.CHN/19.08/1	This study	GQ121433
521-19T/SD/CHN/2007	GenBank	EU753376	EV71/Fuyang.Anhui.CHN/18.08/8	This study	GQ121436
523-05T/SD/CHN/2007	GenBank	EU753397	EV71/Fuyang.Anhui.CHN/19.08/6	This study	GQ121441
523-11T/SD/CHN/2007	GenBank	EU753402			

The average nucleotide homology between Fuyang isolates in this study and those isolated from other provinces in China in 2008, and between Fuyang isolates in this study and the representative sequences from Shandong Province in 2007 Linyi HFMD outbreak were 98.5% and 97.1%, respectively.

### Identification of recombination between HEV71 and CV-A16

**Figure 3:**
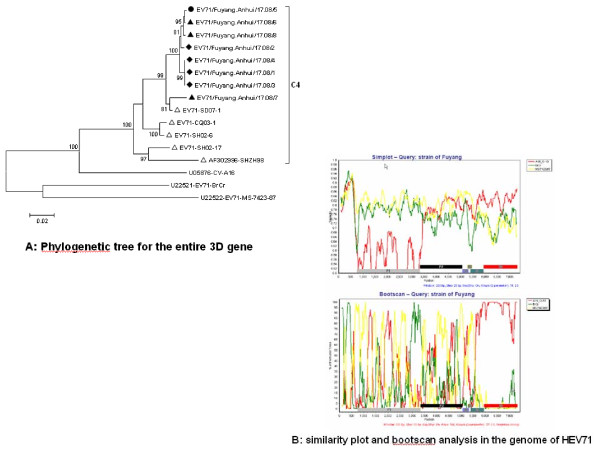
**A: Phylogenetic analysis based on the entire 3D gene**. The trees were drawn on the basis of the 1368-nt sequence including the noncoding region using the Neighbor-Joining method of MEGA software (version 4.0). Solid diamond indicated the fatal cases; Solid triangle indicated the severe cases; Solid round indicated the mild cases; Triangle indicated the HEV71 sequences from other provinces in China between 1997 and 2008(available from GenBank). The scale bar indicates the number of nucleotide substitutions per site. **B: similarity plot and bootscan identified the recombinant sequences in 3D region of genome of HEV71.** The window size of 200-nt slides in increments of 20 nt at a time. The Kimura model with the Jukes-Cantor correction was used. The vertical axis indicates the percent nucleotide identities between strain Fuyang 2008, CV-A16, and HEV71 strain. The horizontal axis indicates the nucleotide positions of the entire genome.

The phylogenetic analysis for 3D region revealed that the sequences of representative isolates from 4 fatal cases, 3 severe cases and 1 mild case shared 77.7%-78.1% and 92.0%-92.2% identities in nucleotides and amino acids, respectively, with the HEV71 prototype strain BrCr; while they shared 84.6-85.0% and 97.4-97.6% identities in nucleotides and amino acids, respectively, with the CV-A16 prototype strain G-10. The phylogenetic tree of the 3D gene showed that the 8 sequences were clustered into the same group and were closer to strain G-10 than to strain BrCr (Figure 3A).

In order to identify the possible recombination events, one of the Fuyang representative strain of HEV71: EV71/Fuyang.Anhui.CHN/17.08/1 was sequenced for the entire genome, and similarity plot and bootscan analysis were used to study the relationship of the Fuyang representative strain of HEV71 to the prototype strains of CV-A16 and HEV71, and genotype B representative strain of HEV71 (Figure 3B). In the analysis, the sequence of the entire genome of the Fuyang strain was used as the query sequence and compared to the sequences of the CV-A16 prototype strain G-10, the HEV71 prototype strain BrCr and B genotype representative strain: MS/7423/87. The results showed that the P1 region of the Fuyang strain had the highest degree of similarity to the sequence of BrCr, and its P2 region and 3A, 3B, 3C region exhibited the highest degree of similarity to the sequence of G-10 and MS/7423/87, while 3D region showed the highest degree of similarity to the sequence of G-10, which suggested that the Fuyang strain might be a recombinant virus with CV-A16 in 3D region.

## Discussion

The diagnosis of virological etiology is critical and challenging at the beginning of the HFMD outbreak. During the Fuyang 2008 outbreak, because the clinical symptoms of HFMD showed atypical manifestations, which was different from common cutaneous lesions, local doctors misdiagnosed HFMD as pneumonia. In addition, the identification of HEV71 infection was hampered by the sudden death of 22 children within 5 days of pulmonary edema or hemorrhage, heart and lung failure. RT-PCR and sequencing were primarily performed to identify the virological etiology of the outbreak. Laboratory testing showed that 39% of the cases were confirmed as HEV71 infection. Together with clinical manifestations and epidemiological data, we concluded that HEV71 was identified as the major etiological pathogen of the Fuyang HFMD outbreak.

In the Fuyang HFMD outbreak, diseases were manifested with cutaneous lesions in mild cases and CNS symptoms in fatal or severe cases. The findings were consistent with studies of the 1997 outbreak in Malaysia and the 1998 outbreak in Taiwan, in which manifestations of HEV71 infections predominate with neurological complications in fatal or severe cases[2,3]. Analyses of the VP1 sequence showed high homology between severe or fatal cases and mild HFMD cases and no significant nucleotide differences were found. Other groups have also studied the complete genome of HEV71 isolated from fatal and non-fatal HFMD cases and found no genetic differences that could be correlated with the severity of clinical manifestation[12,13]. Studies on the pathogenic mechanism for HEV71 have pointed to altered innate immunity. An epidemiologic study during the same outbreak implicated treatment of initial fever of HEV71 infections with glucocorticoids, which block the innate immune response, as contributing to the high rate of severe disease[14]. Experimental animal and clinical studies also support this finding indicating that weakened innate immunity is associated with severe disease and death from HEV71 infection[15-17]. This in combination with introduction of the new recombinant virus may help to explain the increased severity of the Fuyang outbreak.

In this study, genetic recombination event were found between the Fuyang HEV71 isolates, including both fatal cases and non-fatal cases, and CV-A16, which was similar to other studies[18,19]. For enteroviruses[20] and dengue viruses[21], genetic recombination is a well-known phenomenon and recombination could result in the emergence of viruses with altered pathogenic potentials. Except for the Fuyang HEV71 isolates, genetic recombination was also found in the HEV71 viruses, circulated in mainland of China during 1998-2008. However, it was very difficult to clarify the time of recombination and the role of the HEV71 recombinant in HFMD outbreaks remains unclear.

Previous studies showed that all known HEV71 strains could be divided into three distinct genogroups (A, B, C) and 10 subgenogroups (A, B1-5, C1-4) based on VP1 gene sequences; the subgenotype C4 could be further divided into C4a and C4b clusters[8]. Based on the phylogenetic analyses, Fuyang isolates belonged to C4a cluster of the subgenotype C4 and showed high homology with the isolates circulating in other provinces of the mainland of China between 2007 and 2008, including those from the HFMD outbreak in Linyi city in Shandong province in 2007[8]. The C4 subgenotype of HEV71 was initially identified in Guangdong province in 1998 and has been continuously circulating for at least 10 years[10], which may reflect the pattern of endemic circulation of subgenotype C4 viruses. Interestingly, C4b viruses were the predominant circulating strain in mainland of China prior to 2004. Since then, however, C4a viruses have become the predominant strains, which have been continuously circulating and causing epidemic in the mainland of China so far [8].

The subgenotype C4 HEV71 also circulated in neighboring countries and regions, such as, in chronological order, Taiwan, Japan, Vietnam and Thailand[8]; other subgenogroups including B2, B4, B5, C1, C2 co-circulated with C4 between 1990 and 2008[5,7,22]. Interestingly, the subgenotype C4 was the only subgenotype found in China since 1998.

A total of 489,073 HFMD cases, including 126 fatal cases, were reported in China in 2008 (data source: NNDRS). Recombinant HEV71 infection may become more serious public health threat for children under the age of 5 in China, because little is known about the genetics and transmission trend of this fasting mutating virus. To tackle the increasing threat, there is an urgent need for establishing an effective HEV71 surveillance system in China and isolating more viruses, so a complete genetic baseline could be set up for the entire country.

## Conclusion

This study reported the identification of the etiological pathogen of the HFMD ourtbreak in Fuyang city of China, 2008. HEV71 was confirmed to associate with the outbreak. Phylogenetic analyses of entire VP1 capsid protein sequence of 45 Fuyang HEV71 isolates showed that they belong to C4a cluster of the C4 subgenotype. In addition, genetic recombinations were found in the 3D region between the Fuyang HEV71 strain and Coxsackievirus A16 (CV-A16), resulting in a recombination virus. In conclusion, an emerging recombinant HEV71 was responsible for the HFMD outbreak in Fuyang City of China, 2008.

## Methods

### Outbreak investigation

Fuyang city, in the northwest of Anhui Province of China, has 9.76 million inhabitants and a high population density (1,000 per square km). All the cases were reported from local healthcare facility of Fuyang city everyday to National Notifiable Disease Report System (NNDRS) of Chinese Center for Disease Control and Prevention (CCDC). And the number of cases and the incidence rates were taken directly from the reports in the NNDRS in China.

### Clinical specimens and identification

Based on the clinical presentations and case definition, the clinical specimens were collected from fatal cases, severe cases and mild cases individually. From March 1 to May 9, 49 specimens were available from 13 fatal cases, including antemortem specimens (throat swabs, serum, oral efflux), autopsy tissues, lung aspirates, CSF; 122 specimens were collected from 99 severe cases, including throat swabs, stool, and vesicular fluid; 39 throat swabs were from 39 mild outpatients. All patients (or the guardians of the children patients or relatives of fatal or comatose patients) gave oral informed consent. Direct reverse transcription-polymerase chain reaction (RT-PCR) was performed using different sets of primers for panenteroviruses[23]; HEV71[8] and CV-A16[8]. Human rhabdomyosarcoma (RD) and human laryngeal carcinoma (HEp-2) cell lines were used to isolate viruses from clinical specimens. Cultures that exhibited a characteristic enterovirus cytopathic effect were evaluated by RT-PCR and sequencing.

### Sequence determination of the entire VP1 and 3D gene

The entire VP1 and 3D gene of the HEV71 isolates from this outbreak were amplified by RT-PCR with in-house primers that flanked the VP1 gene[8] and 3D gene [HEV71-23S: 5'-ATCACCAAGTTCATACCAGA-3' (nucleotides 5653-5672, relative to strain HEV71/BrCr), and HEV71-28A: 5'-GCTATTCTGGTTATAACAAA-3' (nucleotides 7403-7422, relative to strain HEV71/BrCr)]. The RT-PCR reactions were performed using an Access RT-PCR Kit (Promega, USA). The PCR products were purified using a QIAquick Gel Extraction Kit (Qiagen), and the amplicons were directly sequenced from double direction using an ABI PRISM 3100 Genetic Analyzer (Applied Biosystems, Hitachi, Japan).

### Nucleotide sequence accession numbers

The entire VP1 sequences of 28 representative Fuyang isolates in this study were deposited in the GenBank database under the accession numbers: EU703812-EU703814, GQ121417-GQ121441 (Table 1). And 3D nucleotide sequences of 8 Fuyang isolates were deposited in the GenBank database under the accession numbers: EU703812-EU703814; GQ175176-GQ175180.

### Phylogenetic analysis

Alignment of the entire VP1 and 3D nucleotide sequences of the HEV71 isolates was performed using BioEdit software v5.0.9 (Tom Hall, North Carolina State University, Carolina, USA)[24]. A phylogenetic dendrogram was constructed using MEGA v4.0 program (Sudhir Kumar, Arizona State University, Arizona, USA) [25]. Similarity plot and bootscan analyses for the recombination studies were performed with SimPlot version 3.2[24,26].

## List of abbreviations

HFMD: Hand, foot and mouth disease; HEV71: human enterovirus 71; CV-A16: coxsackievirus A16; NNDRS: national notifiable disease report system.

## Competing interests

The authors declare that they have no competing interests.

## Authors' contributions

YZ, ZZ, WBX prepared manuscript. WBX and DXL designed the study and organized the coordination. YZ, ZZ, XJT performed sequence and data analysis. YZ, ZZ, XJT, NYM, STX, SLZ, ALC, YZ, DMY performed RT-PCR and sequence analysis. WZY, JR, YW, QL, XPD, JZ, YPZ, JFW, ZJF, JLS, SWW collected specimens and performed virus isolation, viral identification. All authors read and approved the final manuscript.
